# Estimation of the Compressive Strength of Corrugated Board Boxes with Shifted Creases on the Flaps

**DOI:** 10.3390/ma14185181

**Published:** 2021-09-09

**Authors:** Damian Mrówczyński, Tomasz Garbowski, Anna Knitter-Piątkowska

**Affiliations:** 1 Research and Development Department, Femat Sp. z o.o., Romana Maya 1, 61-371 Poznań, Poland; damian.mrowczynski@fematproject.pl; 2 Department of Biosystems Engineering, Poznan University of Life Sciences, Wojska Polskiego 50, 60-627 Poznań, Poland; tomasz.garbowski@up.poznan.pl; 3 Institute of Structural Analysis, Poznan University of Technology, Piotrowo 5, 60-965 Poznań, Poland

**Keywords:** corrugated board, box strength estimation, packaging flaps, crease line shifting

## Abstract

In the modern world, all manufacturers strive for the optimal design of their products. This general trend is recently also observed in the corrugated board packaging industry. Colorful prints on displays, perforations in shelf-ready-packaging and various types of ventilation holes in trays, although extremely important for ergonomic or functional reasons, weaken the strength of the box. To meet the requirements of customers and recipients, packaging manufacturers outdo each other with new ideas for the construction of their products. Often the aesthetic qualities of the product become more important than the attention to maintaining the standards of the load capacity of the packaging (which, apart from their attention-grabbing functions, are also intended to protect transported products). A particular flaps design (both top and bottom) and its influence on the strength of the box are investigated in this study. An updated analytical–numerical approach is used here to predict the strength of packaging with various flap offsets. Experimental results indicated a significant decrease in the static load-bearing capacity of packaging in the case of shifted flap creases. The simulation model proposed in our previous work has been modified and updated to take into account this effect. The results obtained by the model presented in this paper are in satisfactory agreement with the experimental data.

## 1. Introduction

The relentless increase in consumption all around the contemporary world is reflected in the significant growth in the production of various goods. This, in turn, entails the necessity of their packing, safe storing and transportation to any destination. Due to growing ecological awareness and concern for the environment, the perfect choice is undoubtedly corrugated cardboard boxes. The undeniable facts are that they are recyclable, easy for disposal, ecological, durable under appropriate conditions and easy to store in a flat form after manufacturing. Among their numerous advantages, one cannot fail to mention the easy imprint of brand names on them. This is highly useful in cases of shelf-ready packaging (SRP) or retail-ready packaging (RRP) when, after being transported to the site, the packaged products are placed directly on the shelves. Upon opening the cardboard boxes along the specially designed and made perforations, products are ready to purchase. Such a solution is a huge time-saver for large companies.

In the case of individual recipients of merchandise, especially when shopping online (which nowadays is a significant part of the sales market), a very important factor is the possibility of smoothly returning purchased products if the consumer is not satisfied, for a range of reasons. Retailers that offer reusable packaging to send back purchased goods are very competitive on the market. Again, corrugated cardboard boxes are perfect in such situations. They are easy to open thanks to well thought-out perforations and, after re-sealing with the built-in adhesive strip, are ready to send back. However, it must not be forgotten that the packaging must have sufficient durability to survive the return transport.

Therefore, in view of the above, scientific research while applying analytical as well as numerical methods and/or laboratory tests has been an inherent part of a separate branch of industry, i.e., the production of corrugated cardboard packaging, for many years.

The proper mechanical strength of the paperboard or corrugated cardboard boxes is directly connected with two characteristic in-plane directions of orthotropy. Machine direction (MD) is perpendicular to the main axis of the fluting and parallel to the paperboard fiber alignment, whilst cross direction (CD) is parallel to the fluting. In order to examine the strength of corrugated cardboard boxes, one can perform some fundamental physical tests, i.e., compressive, tensile or bursting strength tests, which, in practical terms, are the most significant. The most prevalent are the box compression test (BCT) and the edge crush test (ECT) for corrugated cardboard.

A significant impact on the load-bearing capacity of packages is undoubtedly the various perforations, openings and flap locations on corrugated cardboard boxes. The first two issues have been meticulously discussed by Garbowski et al. in [1] and [2], respectively. In the present study, the influence of the flap locations on the strength of corrugated cardboard boxes, as another article in a series, is discussed. The conducting of physical experiments usually involves a great deal of time and cost. Therefore, recently, other methods of testing corrugated boxes have emerged to determine their strength by physical testing only.

Alternatively, the compressive strength of boxes can be assessed based on formulae that have been presented in numerous literatures. Their adoption, thanks to their simplicity, results in quick and easy solutions for practical applications. Moreover, no additional experiments are necessary. The parameters that are introduced in these formulae can be systemized into three groups: paper, board and box parameters [3]. In the first group one can specify: the ring crush test (RCT), Concora liner test (CLT), liner type, weights of liner and fluting, corrugation ratio and a constant related to fluting. In the second one: thickness, flexural stiffnesses in MD and CD, ECT and moisture content. Finally, in the third: dimensions and perimeter of the box, applied load ratio, stacking time, buckling ratio and printed ratio. Nearly 70 years ago, the paper (RCT, flute constant) and box (perimeter, box constant) parameters were applied for the prediction of boxes’ compressive strength in the formula presented by Kellicutt and Landt [4]. The dependence of critical force on paper parameters (CLT, type of liner) and cardboard box dimensions in the BCT was presented in [5].

Generally applicable in the packaging industry is the procedure proposed by McKee et al. [6], in which the parameters of the paperboard (ECT, flexural stiffnesses) and the box perimeter were introduced. Nevertheless, the provided formula is applicable only for comparatively simple boxes. Throughout the years, many scientists endeavored to broaden the applicability of the McKee’s analytical formulae. Allerby et al. [7] modified the constants and exponents in the above-mentioned approach. Schrampfer et al. [8], in turn, amended McKee’s approach by extending the possibility of implementing a broader range of cutting methods and equipment. Batelka and Smith [9] enhanced the relationship with the dimensions of the box and Urbanik and Frank [10] introduced the Poisson’s ratio as well. The arbitrary chosen constant value as a parameter in the McKee’s formula limited its applicability to simple standard boxes. Moreover, Garbowski et al. [1,2,11] examined this approach for more sophisticated cases and modified the McKee’s formula. One cannot forget that the compression strength of corrugated paperboard boxes [12] depends on many factors, such as moisture content of the box [13,14], the presence of openings, ventilation holes and perforations [1,2,15], storage time, stacking conditions [16] and numerous others.

An alternative option to compute the strength of the boxes is to implement, the well-known in engineering, finite element method (FEM). It has been involved in a lot of research, including the problems of numerical analysis with regard to the transverse shear stiffness of corrugated cardboards [17,18,19,20,21] as well as buckling and post-buckling phenomena [22]. The method that efficiently allows one to simplify the examined models is homogenization [23,24,25,26,27]. The result of this procedure is one single layer described by the effective properties of the composite, rather than building the layers made out of different materials. The advantage of this approach to the problem is a significant saving of calculation time while maintaining the appropriate accuracy of the results. The approach based on strain energy, applicable to sandwich panels in the issue of homogenization, was presented by Hohe [28]. For this purpose, a representative element of the heterogeneous and homogenized elements was proposed. Another method, using a periodic homogenization technique considered by Buannic et al. [29], allows not only for an equivalent membrane and the pure bending characteristics of period plates but also, in a modified version, includes the transfer of shear effect in the analysis. The FEM was applied by Biancolini [30] for the examination of a micromechanical part of the considered plate. In the aftermath of application, the energy equivalence between the model and the equivalent plate as well as the stiffness properties of the sandwich plate were obtained. In turn, Abbès and Guo [31] analyzed the plate, which was decomposed into two beams in the directions of the plate, which allowed them to find the torsion rigidity of the orthotropic sandwich plates. The method of treating the quasi-static equilibrium of a material subjected to deformation with hardening was proposed in [32]. Therefore, the experimental data obtained in the dynamic case of deformation could be compared with the data calculated for the quasi-static case. The laboratory tests, properly chosen and scheduled, were performed right on the composite. Layered elements, on which effective parameters can be measured directly, are an alternative method for homogenization. This very approach is proposed in the present research.

An operation during which fold and perforation lines are introduced is defined as creasing. One cannot neglect its impact on the load-bearing capacity of corrugated paperboard. Undeniably, those lines reduce the mechanical strength of the manufactured corrugated paperboard boxes, hence the results of extensive research can be found in the literature. The comparison between the experimental and FEM numerical results, performed in order to examine the creasing influence on the local strength of corrugated paperboard, was discussed by Thakkar et al. [33]. The impact of creasing and subsequent folding on the mechanical properties of laminated paperboard has been picked up by Beex and Peerlings [34], who performed physical as well as numerical experiments, whilst Giampieri et al. [35], to acquire the mechanical response of creased paperboard after folding, used a constitutive model. Domaneschi et al. [36] and Awais et al. [37] proposed an essential (from a practical point of view) solution for the packaging industry, basing it on the FEM simulations of paperboard creasing. Experimental, as well as numerical, studies on the influence of the creasing process during press forming on the paperboard mechanical properties were conducted by Leminen et al. [38].

The particular top and bottom flaps design, which is directly related to the flap creases, and their influence on the strength of corrugated cardboard boxes is investigated in this study. An updated analytical–numerical approach is used to predict the strength of the packaging with various flap offsets. Experimental results pointed out a significant decrease in the static load-bearing capacity of packaging in the case of shifted flap creases. The simulation model, proposed in the previous works of the authors [1,11], has been modified and updated to take this effect into account as well. The results obtained during the analysis of the numerical model proposed in the paper are in adequate agreement with the experimental data. This approach by which the prediction of the strength of boxes with offset flaps is analyzed is, to our knowledge, very pioneering and constitutes an innovative contribution to the development of the field related to the prediction of the load capacity of corrugated cardboard packaging.

## 2. Materials and Methods

### 2.1. Corrugated Board Packaging with Shifted Flaps

**Figure 1:**
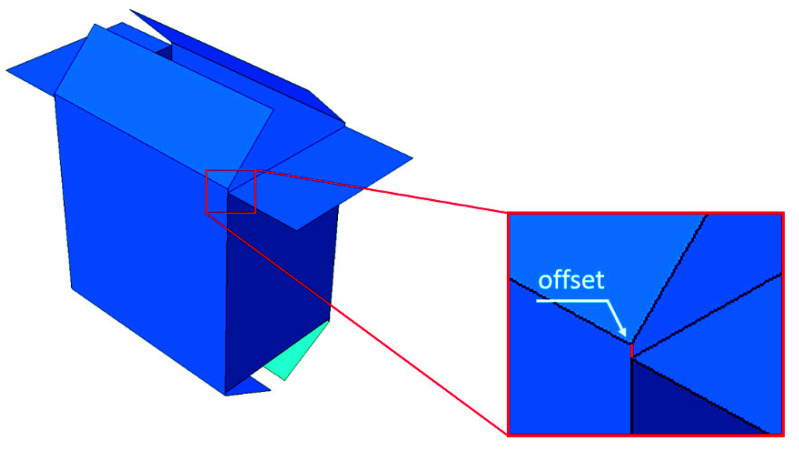
Box with offset flaps.

In previous works, the authors analyzed packages with perforations [1] and openings [2]. Here, the focus is on packages with offset flaps. Such packaging is becoming standard in retail-ready packaging that is also used for shipping. The shifting of the crease line (see Figure 1) makes the flaps more adjustable after closing. Unfortunately, the load-bearing capacity of packaging with shifted flaps significantly diminishes.

**Figure 2:**
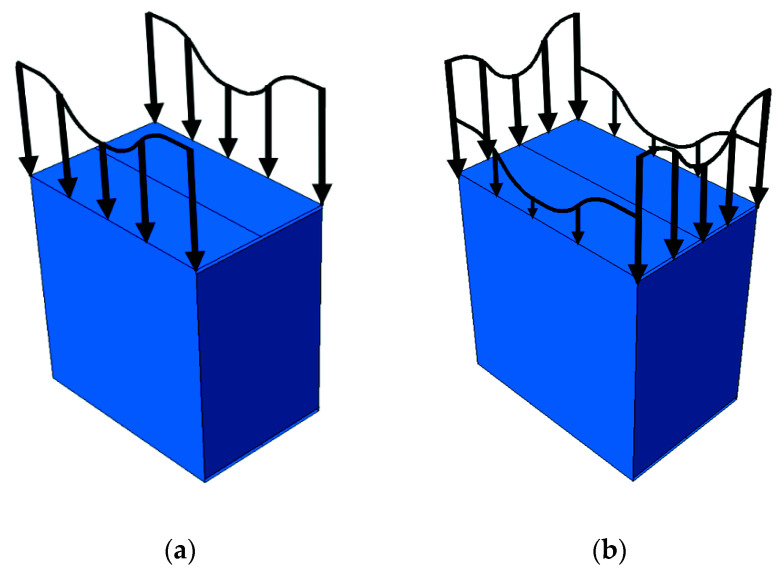
Sequence of loading of the package vertical walls: (**a**) edges loaded in the first step; (**b**) edges loaded in the second step.

The drop in box strength results from a certain sequence of loading, in which the edges of the two shifted (elongated) walls of the package are loaded first, while the other two are only loaded after buckling and/or crushing of the first two (see Figure 2).

**Figure 3:**
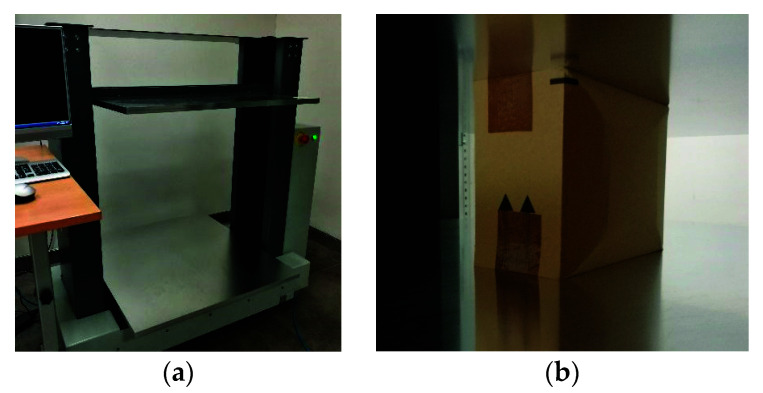
BCT tests: (**a**) BCT press; (**b**) packaging with the shifted offsets on the flaps.

In order to derive a simplified calculation algorithm for estimating the strength of boxes with offset flaps, a series of tests was first performed in the laboratory for various boxes made of different corrugated cardboard. All studies were carried out on the BCT press [39] (see Figure 3). In order to be able to perform computer predictions of the packaging load capacity, it is required in the first step to identify the material parameters of the corrugated board, then to select the appropriate material model and finally to build a numerical or analytical model that takes into account the geometry of the analyzed box.

The following sections describe the laboratory testing of corrugated board, the constitutive modeling of corrugated cardboard, a numerical simulation model and a simple analytical algorithm for estimating the load capacity of corrugated cardboard packaging.

### 2.2. Laboratory Testing of Corrugated Board

Laboratory tests of the corrugated cardboard were performed to determine its stiffness and strength. The four most commonly used tests are: edge crush test, shear stiffness testing, torsional stiffness test and 4-point bending test. The edge crush test (ECT) measures the compressive strength of a corrugated board sample. This test is performed for relatively stocky specimens, so that the failure mechanism is the crushing of the sample, not the loss of stability. The ECT value is often used to determine the load capacity of the corrugated cardboard package in analytical [6], analytical–numerical [1,2,11] or purely numerical [40,41] approaches.

The shear stiffness test (SST) is used to measure the shear stiffness of a sample by applying two equal forces at opposite corners. The measurement of displacements and reaction forces on the supports enables the required stiffness to be calculated. The SST is characterized by a high sensitivity to crushing the sample, resulting in processes such as die-cutting and laminating. The torsional stiffness test (TST) consists of twisting the sample by 10 degrees in both directions and is performed to determine the torsional stiffness. Only the linear part of the bending moment/angle of rotation diagram is being considered for this purpose. The obtained TST values are valid even for highly crushed, broken and flaccid samples.

The bending stiffness test (BNT) is used to determine the bending stiffness in the 4-point bending test. The static scheme of the tested sample allows a constant bending moment and a shear force equal to zero between the internal supports to be obtained, which provides more accurate measurement of the bending stiffness value. On the other hand, the presence of a shear force between internal and external supports makes it possible to take into account the effect of the shear stiffness as well.

### 2.3. Corrugated Board: Material Model and Constitutive Parameters

Since paperboard is an orthotropic material, many material parameters are needed for its correct mathematical description. Therefore, more laboratory tests should be carried out. In papermaking laboratories one can determine visual, functional and mechanical properties of paperboard or corrugated board. The most popular mechanical tests include, for example: (a) short span compression test (SCT) of paperboard; (b) tensile test of paperboard; (c) resistance to bursting of paperboard or corrugated board; (d) edge crush test (ECT) of corrugated board; (e) flat crush test (FCT) of single walled corrugated board; (f) corrugated board bending stiffness (4-point bending test).

**Figure 4:**
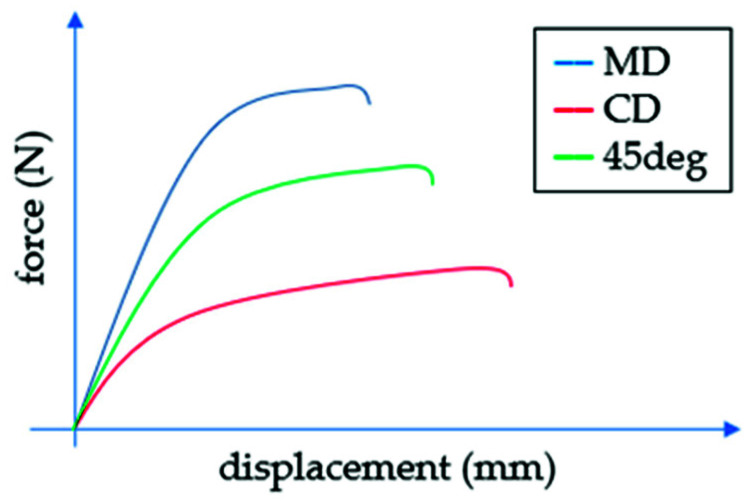
The load–displacement curves in MD, CD and 45 deg.

Some of these tests can be directly used for linear elastic material model calibration, namely the plane strain Young’s modulus in two perpendicular directions, Kirchhoff’s modulus and Poisson’s ratio. The modulus of elasticity (i.e., Young’s modulus) is a quantity well known to designers and engineers, but less common in paper specifications in the cardboard packaging industry. Traditionally, the stiffness modulus can be determined while performing a uniaxial tensile test of a sample. As paperboard is an orthotropic material, more tests are required to determine all elastic parameters (see Figure 4).

Determining the elastic parameters is an important step in the box load-bearing capacity estimation procedure, thus the brief introduction to some basic definitions, the constitutive description of the paperboard and the method of calibrating material constants will be presented in the subsequent sections. For orthotropic materials in a plane stress state, the relationship between elastic strains and stresses can be written as:

(1) $$\left[\begin{array}{c} \varepsilon_{11}\\ \varepsilon_{22}\\ 2\varepsilon_{12} \end{array}\right]=\left[\begin{array}{ccc} 1/E_{1} & -\nu_{21}/E_{2} & 0\\ -\nu_{12}/E_{1} & 1/E_{2} & 0\\ 0 & 0 & 1/G_{12} \end{array}\right]\left[\begin{array}{c} \sigma_{11}\\ \sigma_{22}\\ \sigma_{12} \end{array}\right],$$

where $E_{1}$ is Young’s modulus in the Machine Direction (MD); $E_{2}$ is Young’s modulus in the Cross Direction (CD); $G_{12}$ is Kirchhoff’s modulus and $\nu_{12}$, $\nu_{21}$ are Poisson’s coefficients. Due to the symmetry of the material compliance/stiffness matrix, the relationship between the Poisson’s coefficients is as follows:

(2) $$\frac{\nu_{12}}{E_{1}}=\frac{\nu_{21}}{E_{2}}.$$

The Hill model [42] can be successfully employed to describe the behavior of the paper in an inelastic phase. Implementation of the Hill model requires the definition of the elastic domain described by the plastic yield function and the description of the material hardening:

(3) $$f\left(\sigma ,\kappa \right)=\sqrt{a_{1}\sigma_{11}^{2}+a_{2}\sigma_{22}^{2}-a_{12}\sigma_{11}\sigma_{22}+3a_{3}\sigma_{12}^{2}}-\sigma_{0}\left(\kappa \right)\leq 0,$$

where $\sqrt{*}$ is an effective stress $\sigma_{eff}$, which can be reduced to classical Huber-Mises criterion for isotropic materials if $a_{1}=a_{2}=a_{12}=a_{3}=1$; $\sigma_{0}\left(\kappa \right)$ is a yield stress function; κ is a hardening parameter, usually related to effective plastic strains; $\sigma_{ij}$ are the stresses in main orthotropic directions; $a_{i}$ and $a_{12}$ are called anisotropic parameters, which can be determined from simple tensile tests in the main orthotropic directions:

(4) $$a_{1}=\frac{\sigma_{0}^{2}}{\sigma_{10}^{2}},\;\;\;\;\;a_{2}=\frac{\sigma_{0}^{2}}{\sigma_{20}^{2}},\;\;\;\;\;a_{3}=\frac{\sigma_{0}^{2}}{3\sigma_{120}^{2}},$$

where: $\sigma_{0}$ is the initial yield stress in the reference direction; $\sigma_{10}$ is the yield stress in first direction (e.g., MD); $\sigma_{20}$ is the yield stress in second direction; $\sigma_{120}$ is the yield stress in shearing.

The remaining parameter $a_{12}$ can be determined from the equation:

(5) $$a_{12}=a_{1}+a_{2}+3a_{3}-4a_{45},$$

where $a_{45}$ is the anisotropic parameter determined from a tensile test in an angled direction of 45 deg. As for most materials, only the values $\sigma_{10}\;$, $\sigma_{20}$ and $\sigma_{120}$ are known, in practical applications for the coefficient $a_{12}$ usually a simplified relationship is assumed, e.g.,:

(6) $$a_{12}=\frac{\sigma_{0}^{2}}{\sigma_{10}\sigma_{20}}.$$

It is a known fact that paperboard behaves differently under tension and compression. Therefore, the chosen plasticity criterion (which is symmetric in case of tension and compression) is not appropriate for this type of material. However, for simple strength calculations with a stress state dominated by compression, this model is a sufficient approximation. For the correct analysis of the structure in the complex stress state, one of the more sophisticated constitutive models should be used, e.g., [43,44,45,46,47,48].

### 2.4. Numerical Predictive Model

**Figure 5:**
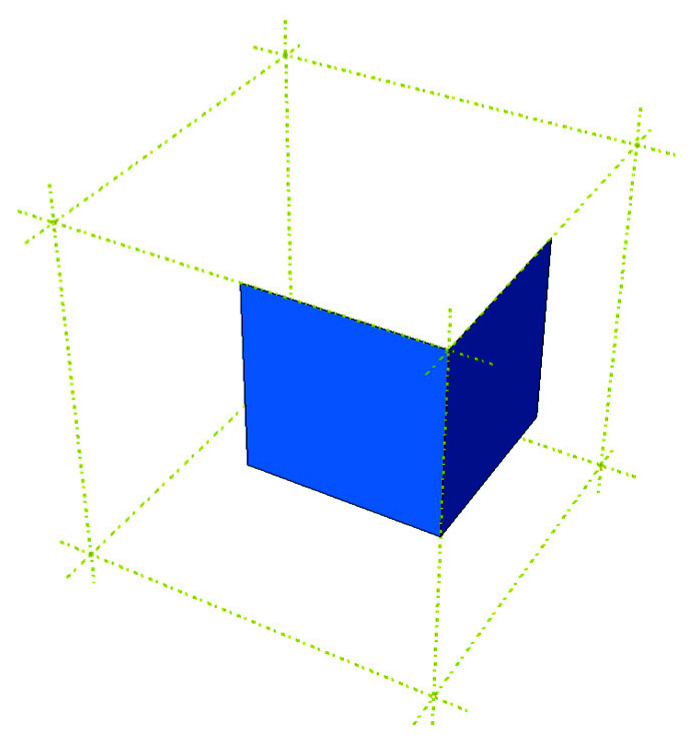
Scheme of the 1/8 part of the package.

The numerical model of the box was built in the Abaqus Unified FEA software (2020, Dassault Systèmes Simulia Corp., Providence, RI, USA.) [49]. Two types of models had to be created: (i) the non-offset packaging and (ii) the package with flaps offset. In order to simplify the computations and save the computing time, only 1/8 part of the box was modeled instead of the whole packaging (see Figure 5). The material used in the model was linear elastic orthotropic model with Hill plasticity.

**Figure 6:**
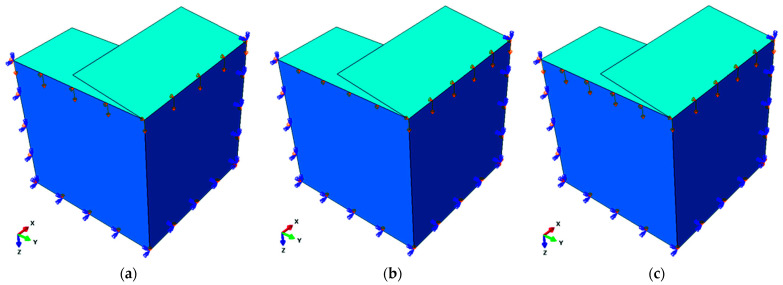
Boundary conditions for the case of: (**a**) the non-offset package; (**b**) the package with offset flaps (first step); (**c**) the package with offset flaps (second step).

To obtain the appropriate behavior of the numerical model, symmetry boundary conditions were defined on each edge (see Figure 6). For the packaging model without offset, only one computation step was defined, in which the displacement was applied on both edges. In the case of the package with offset flaps, in the first step only the offset edge was loaded and in the second step the load was then applied to the non-offset edge. The 4-node quadrilaterals shell elements with full integration, named S4, were used for all computations. For different dimensions of packaging, different values of mesh size were assumed. For example, for the package dimensions 500 × 500 × 500 mm the approximate global size of the element was 12 mm, which ultimately gave 882 elements, 946 nodes and 5676 degrees of freedom. To add the initial deformations (resulting from imperfections) of the box vertical walls, a buckling analysis was performed before the main calculations. The first buckling mode of the model found in this way was later introduced in the next step in the form of scaled imperfections.

### 2.5. Analytical Predictive Model

The simplified procedure for estimating the compressive strength of a corrugated cardboard box with offset flaps proposed here is based on an analytical model. The algorithm exploits the basic constitutive parameters of a single box wall, namely: $ECT_{CD}$—compressive strength in CD, $E_{CD}=E_{2}$—compressive stiffness of corrugated boards in CD and $E_{MD}=E_{1}$—compressive stiffness of corrugated boards in MD. Since in some cases the instability of a single wall may occur before plasticization, it is also necessary to determine the critical load for an orthotropic rectangular plate, e.g., from the formula [1,2,11]:

(7) $$P_{cr}^{i}=\frac{\pi^{2}}{B_{i}{}^{2}}\frac{t_{i}{}^{3}}{12}\sqrt{E_{CD}^{i}E_{MD}^{i}}{\left(\frac{mB_{i}}{H}+\frac{H}{mB_{i}}\right)}^{2},$$

where $B_{i}$ is the width of the *i*-th panel; $t_{i}$ is the *i*-th panel thickness; H is the box height; m is the number of half-waves for which $P_{cr}^{i}$ reaches the minimum.

The analysis of strength estimation of a box with shifted flaps, as already discussed in the previous section, consists of two stages, in which the higher walls (i.e., the shifted ones) are loaded first (see Figure 2a), while the lower walls are loaded only if preliminary crushing and/or buckling of the first two walls occurs (see Figure 2b). Therefore, the overall load capacity of the packaging is the sum of the load capacity of two pairs of opposite walls of the box, namely:

(8) $$BCT=\alpha BCT_{1}+BCT_{2},$$

where

(9) $$BCT_{1}=2kECT^{r}{\left(P_{cr}^{1}\right)}^{1-r}\gamma_{1}\gamma_{2}B_{1}$$

is the load capacity of the shifted walls, while

(10) $$BCT_{2}=2kECT^{r}{\left(P_{cr}^{2}\right)}^{1-r}\gamma_{3}\gamma_{4}B_{2}$$

is the load capacity of lower walls.

**Figure 7:**
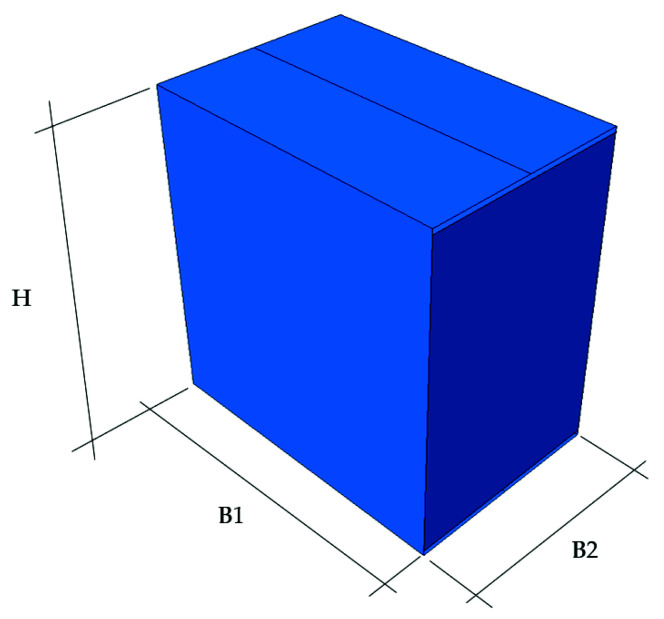
Box dimension symbols.

**Figure 8:**
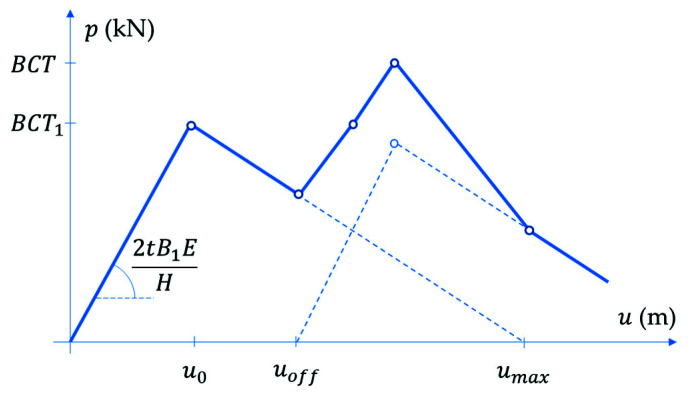
Force-displacement visualization of the proposed method.

In Equations (9) and (10) k is a certain constant and r is an exponent, r∈(0,1), and $\gamma_{i}$ are the reduction coefficients. $B_{1}$ and $B_{2}$ are base dimensions, which are shown in Figure 7. The α coefficient reduces the value of the first term in Equation (8) due to the initial failure and/or buckling of the walls loaded in the first step (see Figure 8). This factor can be calculated using the formula below:

(11) $$\alpha =1-\frac{u_{off}-u_{0}}{u_{max}-u_{0}},$$

where $u_{off}$ is an offset of higher walls; $u_{max}=H$ is assumed to be equal to the height of the box; $u_{0}$ is the vertical deformation corresponding to the maximum load. The latter can be calculated from Hooke’s law considering the stiffness in the CD direction, $E_{CD}$; single box wall height, H (see Figure 7); shifted wall width, $B_{1}$; board thickness, t; the compressive strength, $BCT_{1}$ (see Figure 8). Thus, finally we obtain:

(12) $$u_{0}=\frac{BCT_{1}}{2tB_{1}E_{CD}}H.$$

The reduction factors $\gamma_{i}$ are always less than one and depend on the ratio of the box dimensions and the exponents $r_{i}$. The $\gamma_{1}$ factor in Equation (9) reads:

(13) $$\gamma_{1}=\min\left[{\left(\frac{B_{1}}{H}\right)}^{r_{1}},\;1\right],$$

while $\gamma_{2}$:

(14) $$\gamma_{2}=\min\left[{\left(\frac{B_{1}}{B_{2}}\right)}^{r_{2}},\;1\right].$$

Similarly, the coefficient $\gamma_{3}$ in Equation (10) is:

(15) $$\gamma_{3}=\min\left[{\left(\frac{B_{2}}{H}\right)}^{r_{3}},\;1\right],$$

while $\gamma_{4}$:

(16) $$\gamma_{4}=\min\left[{\left(\frac{B_{2}}{B_{1}}\right)}^{r_{4}},\;1\right].$$

All unknown factors in Equations (8)–(10), namely constant k and exponent r, and the four exponents $r_{i}$ in Equations (13)–(16), can be found by calibration with experimental data. The calibration procedure will be presented in the following section.

### 2.6. Calibration Procedure

The main goal of this study is to propose a reliable analytical model for the quick estimation of the load capacity of offset packaging. Therefore, the calibration of the coefficients in the analytical equations is particularly important. Unfortunately, the limited number of laboratory results creates a risk that the analytical model will be valid only for a small set. In order to extend the applicability of the proposed model, a calibration procedure consisting of two stages was engaged: (i) in the first step, special attention was paid to the correct mapping of experimental results into a numerical model; (ii) in the second one, the already tuned numerical model was used to generate much larger sets of cases, which were then utilized to identify the sought parameters in the analytical model.

In the first step, the only unknowns are the initial imperfections. Therefore, a very simple strategy is used, in which the numerical model is calibrated with experimental data by appropriate scaling of the initial deformations of the vertical box walls. In the second step, the coefficients in Equations (9) and (10) are identified in the assumed order: first the constant k as well as exponents r and $r_{1}$, then $r_{2}$ and $r_{3}$. In both cases, simple techniques were used to minimize the discrepancy between analytical model prediction and numerical results with the use of the least squares method.

## 3. Results

### 3.1. Corrugated Board: Material Testing

In order to correctly determine the properties of the material, it was necessary to examine samples of corrugated board in several typical laboratory tests. For this purpose, a FEMat BSE device (FEMat Sp z o.o., Poznan, Poland) [50] was used. In total, seven different types of corrugated cardboard with a grammage of 350 to 965 g/m^2^ were tested. Since cardboard is a very heterogeneous material, at least 10 samples in each test were examined for each grade in order to obtain statistically reliable results. In Table 1, the sample results for the BC-780 grade are summarized. The first column represents a test number, the second column shows the sample thickness and in the third to ninth columns the results obtained from different tests in both orthotropy directions are demonstrated (all test symbols are explained in the previous section).

**Figure 9:**
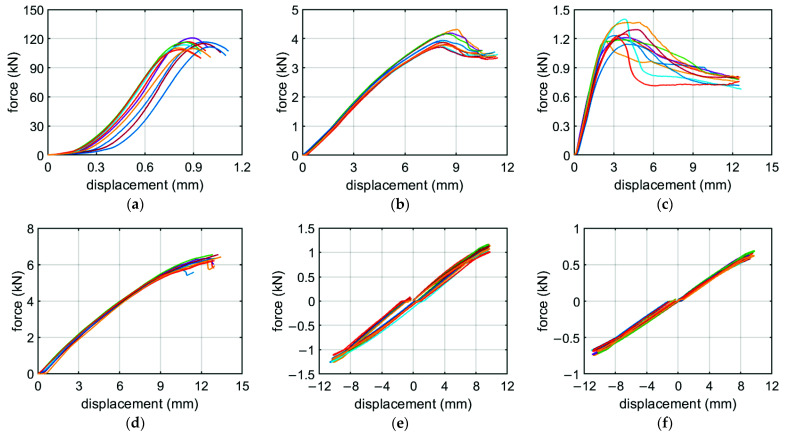
Force-displacement curves for BC-780 corrugated cardboard in various tests: (**a**) ECT; (**b**) BNT–MD; (**c**) BNT–CD; (**d**) SST; (**e**) TST–MD; (**f**) TST–CD.

Figure 9 demonstrates the force-displacement curves from all tests of the corrugated cardboard. Since both shape of the curve and the calculated shear stiffness (SST) in the machine and cross directions are almost identical, only the values in the MD are shown. In Table 2, the mean values of the tests for all seven grades are presented. The first column represents grades that were used in the packaging for which the box compression test was carried out (details will be discussed in the next section). In the second to ninth columns, the measured stiffnesses obtained from the BSE device are shown.

### 3.2. Box Compression Test (BCT)

In the next step, the load capacity of the packaging was checked. For this purpose, the FEMat BCT-20T20 compact press (FEMat Sp. Z o.o., Poznan, Poland) [38] was exploited (see Figure 3a). A total number of 18 samples of various dimensions and materials were prepared. The analysis was carried out for two types of packaging: without and with an offset. In Table 3, the results obtained with the box compression test are presented. In the first column, corrugated cardboard grades are shown. The second, third and fourth columns show the dimensions of the package (see Figure 7). For offset packaging, the edge of the $B_{1}$ dimension is the offset edge. The fifth column represents the value of the load capacity of the package without offset. Columns six and seven are the BCT values for the offset package: the sixth column is the value of the first extreme and the seventh column is the value of the second extreme.

**Figure 10:**
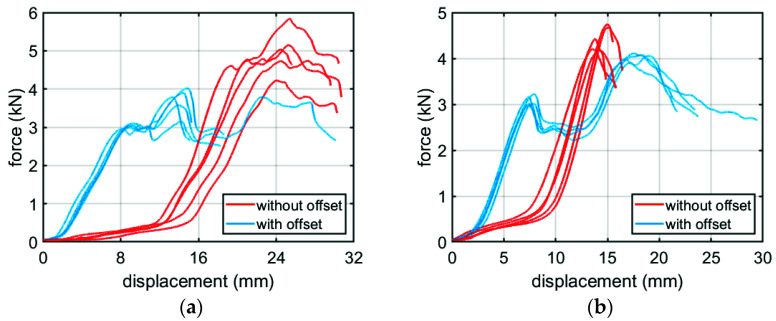
Selected measurements from a BCT press for grades: (**a**) BC-780; (**b**) EB-965.

In Figure 10, the force-displacement diagrams for boxes with dimensions 300 × 200 × 200 mm, with and without offset, made of BC-780 and EB-965 corrugated cardboard are shown.

### 3.3. Prediction Results of the Numerical Model

Having the geometry of all the tested boxes and the material properties of the corrugated cardboards, it was possible to build numerical models and calibrate the only one remaining component: the initial imperfections. These are especially important in the geometrically nonlinear FE analysis. To introduce preliminary deformations into the model, first a buckling analysis was carried out to find the first preferred buckling mode, which was then introduced as a deformed shape of the load-bearing panels of the box.

**Figure 11:**
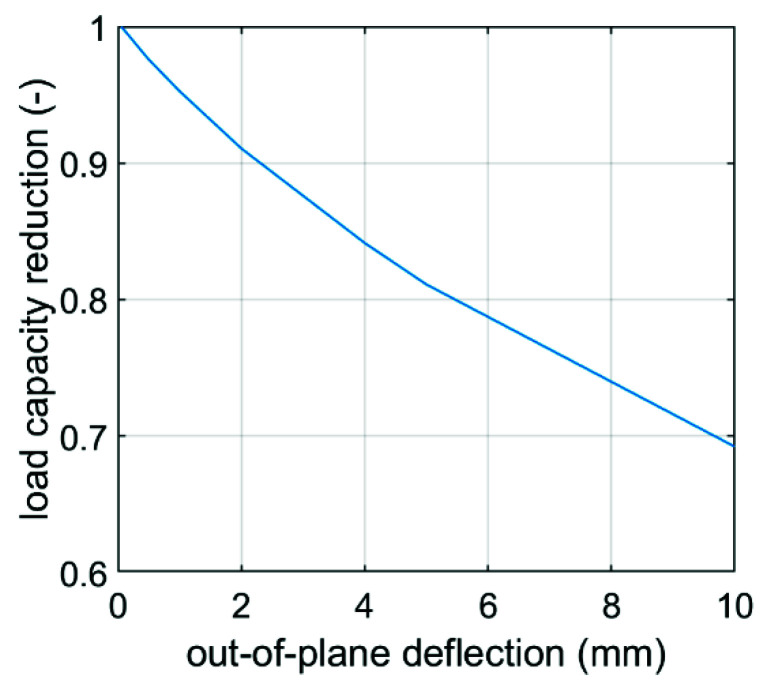
Influence of the imperfections on the load capacity of the BC-780 box.

The influence of the imperfection size on the load capacity of the box 300 × 200 × 200 mm made of BC-780 is shown in Figure 11.

After a successful calibration procedure, the results obtained with the numerical model are summarized in Table 4, which also shows the differences between the calculated and the measured values of the BCT.

### 3.4. Prediction Results of the Analytical Model

As already discussed, the main step was to calibrate the coefficients in the analytical formulas for the load capacity estimation of corrugated board packaging. For this purpose, synthetically generated results were utilized. Thanks to the use of numerical results, the range of packaging dimensions was much wider, which resulted in a greater number of analyzed cases and therefore made the calibration more reliable.

**Figure 12:**
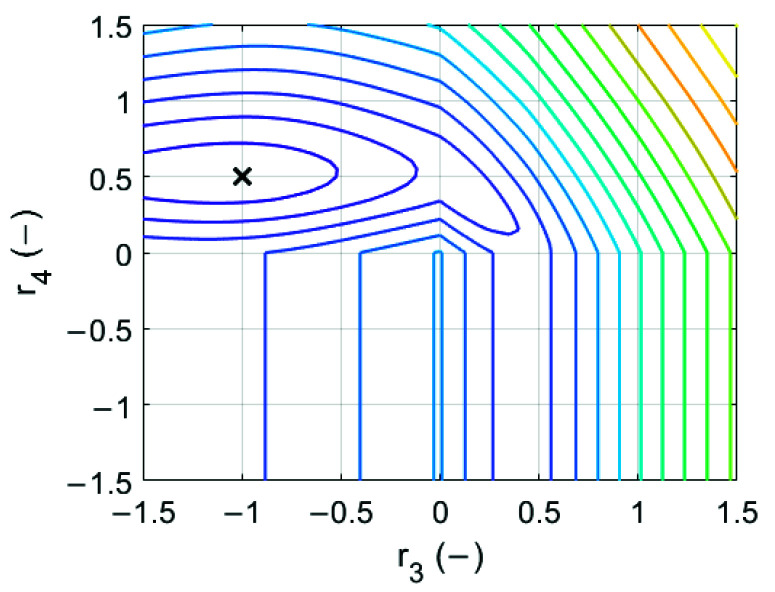
Error function in the sought parameters ($r_{3}$ and $r_{4}$) space. The location of the optimal value is marked with ‘x’.

Table 5 shows all coefficients found in the minimization process used in Equations (9) and (10), while in Figure 12 the discrepancy function in two-dimensional space $\left[r_{3},r_{4}\right]$ is shown. It can be seen that in the selected range of parameters $r_{3}$ and $r_{4}$ there is only one local minimum, which is also the global minimum (see Figure 12).

**Figure 13:**
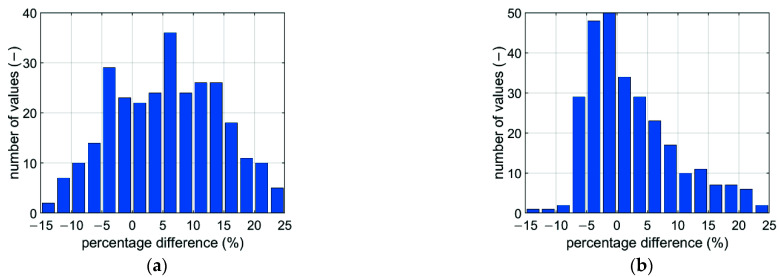
The prediction error distribution obtained while using the analytical model: (**a**) first extreme; (**b**) second extreme.

Figure 13 shows the estimation errors obtained from the analytical model in the calibration procedure for all offset and non-offset boxes. Table 6 presents a comparison of the results obtained from the tuned analytical model with the experimental results.

**Figure 14:**
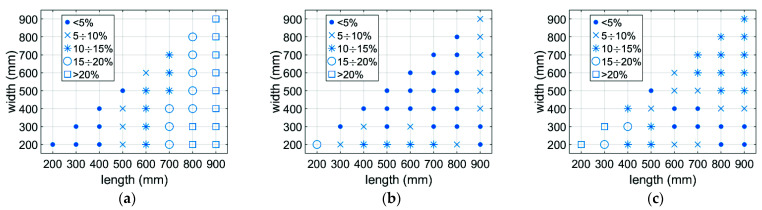
The prediction error distribution obtained using the analytical model for the first extreme: (**a**) H = 200 mm; (**b**) H = 500 mm; (**c**) H = 800 mm.

**Figure 15:**
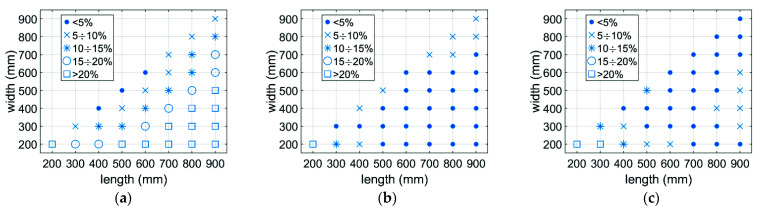
The prediction error distribution obtained using the analytical model for the second extreme: (**a**) H = 200 mm; (**b**) H = 500 mm; (**c**) H = 800 mm.

Figure 14 and Figure 15 show the distribution of the prediction error in the design space, which are the main dimensions of the box (L, B, H). It can be seen that the greatest error occurs with boxes that are short and long.

## 4. Discussion

Since corrugated board is an orthotropic and non-homogeneous material, a large number of tests were required for the correct characterization of its mechanical parameters. This means that when testing both corrugated cardboard and boxes made of such material, one can expect a large dispersion of test results. This is related to the heterogeneity of the paper itself, as well as the corrugated cardboard, and the inaccuracy of the assemblies of the tested packaging. Thus, the number of tests in the case of boxes should not be less than five, as is the case with testing the corrugated cardboard samples.

Among the many mechanical tests of corrugated board available in the papermaking laboratory, the most important define not only the static edge crush resistance but also the flexural and torsional stiffness of the specimen. In this study, the BSE system [50], which allows the examination of five physical parameters of cardboard (for three of them in both directions of orthotropy), was exploited. Based on the results of all laboratory tests (see Table 1 and Figure 9), homogenized parameters describing the elastic and plastic behavior of the particular corrugated cardboard were obtained.

In order to diversify the set of BCT laboratory results, various corrugated cardboards (a total of seven types) that are used for box production and nine different dimensions of the packaging structure, in two variants (without and with offsets), were tested. The results are presented in Table 3, where among the dimensions of the boxes and the symbols of the corrugated board one can also find the BCT results for two cases: (a) without offsets in column five and (b) with offsets in columns six and seven. The sixth column of Table 3 presents the force value for which two offset walls have been crushed. This is clearly seen in Figure 10: the first peak in the blue result plots. Column seven of Table 3 shows the maximum force value obtained in the BCT test.

Both predictive models take into account the behavior shown in Figure 10, which is characteristic for the offset boxes. The numerical model is loaded sequentially, first on the walls with an offset and then when the displacement of the upper surface exceeds the given offset, the two remaining walls are also loaded. As already mentioned, after calibrating the material model and for the given geometry, the only unknown was the size of the imperfections of the vertical walls. These parameters were in each case adjusted so that the estimates agreed with the laboratory results. The effect of the applied imperfection in a specific case (box EB-780) is shown in Figure 11.

This phenomenon is treated slightly differently in the analytical model, which is based solely on the geometry of the box, its strength in CD and both stiffnesses in MD and CD. In this case, the imperfections are embedded in the predictive model through the critical load term in Equations (9) and (10), while the sequential crushing of the shifted and non-shifted faces is captured by independently determining two values and scaling the maximum force in the first peak by the factor α (see Equation (8)). This allows the degraded resistance of walls with an offset and the resistance of walls without an offset to be taken into account in the second peak. The tuning exponents found by the minimization procedure (shown in Table 5) reached the optimal values of 0.5 or 1.0, while the constant k and exponent r reached values of 0.75 and 0.55, respectively.

The use of data synthetically generated by the calibrated numerical model allowed a much greater accuracy of the tuned parameters in the analytical model to be obtained. This was mainly due to a larger range of results numerically generated for various geometric dimensions of boxes that could not be physically produced and tested in the BCT press. Figure 13 shows the distribution of the prediction error of the load capacity of various corrugated board packages without and with an offset. The largest discrepancies occur for packages with a relatively large proportion of dimensions (see Figure 14 and Figure 15). However, the average error in both cases does not exceed 7%. Overall, the proposed predictive analytical model can capture the first peak in any experimentally tested sample fairly correctly, and the error in most cases is less than 7%. The greatest differences can be observed for samples B-400-1 and B-400-2, where the error was 8% and 9%, respectively. Similar conclusions can be drawn when predicting a second peak. In most cases, the error did not exceed 5%; only for the B-400-2 sample did it reach 9%.

In general, the application of analytical models existing in the literature, e.g., those proposed in [4,5,6,7,8,9,10] or even more the recent models presented by Garbowski et al. [1,2,11], does not allow one to predict the strength of boxes with shifted flaps. The reason is that these models do not take into account the sequential crush of the package walls. Particular attention should also be paid to modeling with purely numerical models, because special techniques for sequential loading of the walls with appropriate imperfections should be considered as well. The results presented in Table 4 and Table 6 show the precision with which both the numerical model and the proposed analytical model reflect the laboratory results for selected constructions of corrugated cardboard boxes. The results obtained from both models do not differ by more than 10% from the experimental results.

## 5. Conclusions

This article presents numerical and analytical models for predicting the strength of boxes with displaced flaps. The obtained results are in accordance with the conducted laboratory tests. In both models, the mechanical parameters of the corrugated board obtained from the selected laboratory tests were implemented. Both models are based on a sequential approach for the loading of the vertical walls of a box; the walls with an offset are loaded first, then the walls without an offset. At the moment of loading the walls without offset, the two walls loaded in the first step are already partially damaged. Therefore, this type of packing is characterized by much lower load-bearing capacity than packages with flaps without an offset. Thanks to the methodology presented in this paper and utilization of such predictive tools, it is possible not only to design packaging more consciously, but also to deliver and optimally use the material for their manufacturing, and thus improve the sustainable economy of the production plant.

## Figures and Tables

**Table 1:** Test values for BC-780 corrugated cardboard grade.

Test	THK	ECT	BNT-MD	BNT-CD	SST-MD	SST-CD	TST-MD	TST-CD
1	6.49	10.77	10.79	10.47	2.96	3.05	3.10	1.79
2	6.50	10.66	10.55	9.66	3.02	2.77	3.05	1.74
3	6.49	10.93	10.53	9.20	2.90	2.99	3.08	1.79
4	6.53	11.28	10.31	10.11	2.80	2.86	3.26	1.71
5	6.53	11.15	10.29	11.24	2.95	2.91	3.20	1.70
6	6.52	11.41	11.13	11.94	2.95	2.77	3.31	1.92
7	6.52	11.85	11.06	10.92	2.95	2.77	3.29	1.85
8	6.55	10.82	11.11	11.03	2.96	2.70	3.29	1.90
9	6.53	11.44	10.42	9.05	3.10	2.90	3.45	1.88
10	6.55	11.44	10.74	10.43	3.12	2.87	3.35	1.88

**Table 2:** Test values for corrugated cardboard grades.

Grade	THK	ECT	BNT-MD	BNT-CD	SST-MD	SST-CD	TST-MD	TST-CD
E-350	1.49	4.68	0.36	0.80	0.19	0.24	0.18	0.18
E-380	1.59	5.41	0.49	1.16	0.26	0.31	0.23	0.23
B-400	2.80	5.50	1.50	2.94	0.55	0.57	0.60	0.38
EE-585	2.77	9.05	1.46	2.94	0.67	0.71	0.70	0.73
BC-780	6.52	11.18	10.69	10.41	2.97	2.86	3.24	1.82
EB-880	4.42	15.11	6.32	10.70	2.33	2.28	2.47	2.06
EB-965	4.55	13.69	5.68	11.39	2.24	2.26	2.42	1.89

**Table 3:** Main dimensions and BCT values of various corrugated cardboard packaging.

Name	$B_{1}$	$B_{2}$	H	BCT (N)		
	(mm)	(mm)	(mm)	Without Offset	With Offset 1	With Offset 2
E-350-1	300	200	300	875	566	767
E-350-2	450	100	450	704	454	656
E-380	300	200	300	1003	663	1131
B-400-1	300	200	300	2048	1265	1556
B-400-2	450	100	450	1498	1104	1201
EE-585	300	200	300	2409	1452	1855
BC-780	300	200	200	4995	2989	3817
EB-880	300	200	300	5352	3404	3700
EB-965	300	200	200	4445	3124	3830

**Table 4:** Comparison of measured and numerically determined BCT values for various corrugated cardboard packaging.

Name	BCT (N)			
	Measured Values		Numerical Values	
	First Extreme	Second Extreme	First Extreme	Second Extreme
E-350-1	566	767	520	778
E-350-2	454	656	448	648
E-380	663	1131	641	1132
B-400-1	1265	1556	1185	1540
B-400-2	1104	1201	1126	1117
EE-585	1452	1855	1468	1834
BC-780	2989	3817	2993	3690
EB-880	3404	3700	3222	3555
EB-965	3124	3830	3265	3653

**Table 5:** Coefficients values.

k	r	$r_{1}$	$r_{2}$	$r_{3}$	$r_{4}$
	0.55	0.50	1.00	−	−
0.75	0.55	−	−	−1.00	0.50

**Table 6:** Comparison of measured and analytically determined BCT values for various corrugated cardboard packaging.

Name	BCT (N)			
	Measured Values		Analytical Values	
	First Extreme	Second Extreme	First Extreme	Second Extreme
E-350-1	566	767	553	752
E-350-2	454	656	471	657
E-380	663	1131	709	1135
B-400-1	1265	1556	1171	1642
B-400-2	1104	1201	1197	1323
EE-585	1452	1855	1516	1913
BC-780	2989	3817	2975	3764
EB-880	3404	3700	3360	3854
EB-965	3124	3830	3079	3877

## Data Availability

The data presented in this study are available on request from the corresponding author.
